# Monthly Distribution of Phlebotomine Sand Flies, and Biotic and Abiotic Factors Related to Their Abundance, in an Urban Area to Which Visceral Leishmaniasis Is Endemic in Corumbá, Brazil

**DOI:** 10.1371/journal.pone.0165155

**Published:** 2016-10-26

**Authors:** Everton Falcão de Oliveira, Aline Etelvina Casaril, Wagner Souza Fernandes, Michelle de Saboya Ravanelli, Márcio José de Medeiros, Roberto Macedo Gamarra, Antônio Conceição Paranhos Filho, Elisa Teruya Oshiro, Alessandra Gutierrez de Oliveira, Eunice Aparecida Bianchi Galati

**Affiliations:** 1 Programa de Pós-Graduação em Saúde Pública, Faculdade de Saúde Pública, Universidade de São Paulo, São Paulo, SP, Brasil; 2 Laboratório de Parasitologia Humana, Centro de Ciências Biológicas e da Saúde, Universidade Federal de Mato Grosso do Sul, Campo Grande, MS, Brasil; 3 Agência Estadual de Defesa Sanitária Animal e Vegetal, IAGRO, Corumbá, MS, Brasil; 4 Departamento de Estatística, Campus Macaé, Universidade Federal do Rio de Janeiro, Macaé, RJ, Brasil; 5 Laboratório de Geoprocessamento para Aplicações Ambientais, Faculdade de Engenharias, Arquitetura e Urbanismo e Geografia, Universidade Federal de Mato Grosso do Sul, Campo Grande, MS, Brasil; 6 Departamento de Epidemiologia, Faculdade de Saúde Pública, Universidade de São Paulo, São Paulo, SP, Brasil; Fundacao Oswaldo Cruz, BRAZIL

## Abstract

The monthly distribution and abundance of sand flies are influenced by both biotic and abiotic factors. The present study aimed to evaluate the seasonal distribution of sand flies and the relation between their abundance and environmental parameters, including vegetation and climate. This study was conducted over a 2-year period (April 2012 to March 2014). Monthly distribution was evaluated through the weekly deployment of CDC light traps in the peridomicile area of 5 residences in an urban area of the municipality of Corumbá in the State of Mato Grosso do Sul, Brazil. Meteorological data were obtained from the Mato Grosso do Sul Center for Weather, Climate, and Water Resources. The spectral indices were calculated based on spatial resolution images (GeoEye) and the percentage of vegetal coverage. Differences in the abundance of sand flies among the collection sites were assessed using the Kruskal-Wallis test, and the strength of correlations between environmental variables was determined by calculating Spearman’s correlation coefficients. *Lutzomyia cruzi*, *Lu*. *forattinii*, and *Evandromyia corumbaensis* were the most frequently found species. Although no significant association was found among these sand fly species and the tested environmental variables (vegetation and climate), high population peaks were found during the rainy season, whereas low peaks were observed in the dry season. The monthly distribution of sand flies was primarily determined by *Lu*. *cruzi*, which accounted for 93.94% of the specimens collected each month throughout the experimental period. The fact that sand flies were detected year-round indicates a continuous risk of infection to humans, demonstrating the need for targeted management and education programs.

## Introduction

The monthly distribution and abundance of sand flies is influenced by both biotic and abiotic factors. Temperature, humidity, and rainfall exert a direct influence on sand fly populations, with effects being dependent on the region, weather, and species analyzed [1–4].

Geospatial tools, geographic information systems (GIS), and geostatistics have facilitated studies on how health, the environment, and socioeconomic conditions are related with the temporal and spatial distributions of different diseases and vector populations [2,5–8]. Such studies have provided important information for health surveillance, providing data for monitoring and mapping risk factors, as well as providing better descriptions, understanding, and predictions of geographic distribution [5,7,8].

Different spectral indices, which are calculated from the relationship of different bands of satellite images, allow us to obtain specific information about land cover, such as the presence of urbanized areas or bodies of water, vegetal coverage, and leaf area [9,10]. Use of data obtained from satellite images, such as the normalized difference vegetation index (NDVI), has allowed the identification and monitoring of vegetation diversity, as well as the determination of geographical space and areas at risk of endemic diseases, such as visceral and cutaneous leishmaniasis, and how they affect vector populations [2,6,7,11,12].

In South America, the spatial distribution pattern of *Lutzomyia longipalpis* is positively associated with the presence of vegetation in the peridomestic environment [3,13]. In Europe, Asia, and Africa, weather data and remote sensing have been used to predict the geographic and seasonal distribution of *Phlebotomus* spp., with these spatial variables being strongly correlated with species presence [11,12,14].

This study aimed to evaluate the seasonal distribution of sand flies, as well as to investigate possible associations between the most abundant species and environmental variables related to vegetation and climate.

## Materials and Methods

### Study Area

**Fig 1:**
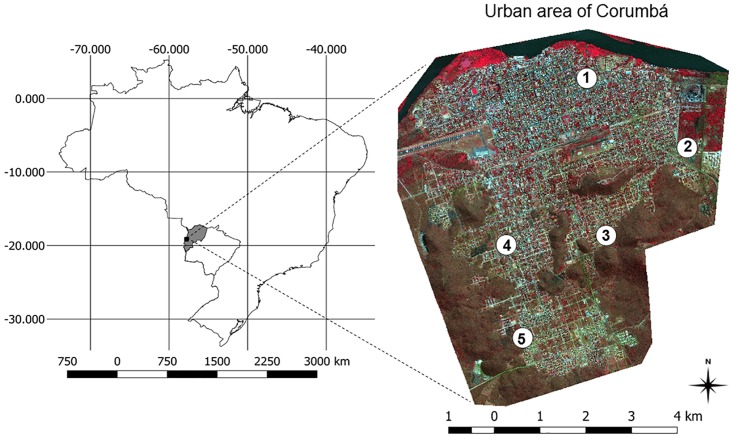
Spatial distribution of sand fly sampling sites in the urban area of Corumbá, Mato Grosso do Sul, Brazil. Numbers 1–5 indicate sand fly sampling sites (neighborhoods): 1 = Center; 2 = Maria Leite; 3 = Cristo Redentor; 4 = Popular Nova; 5 = Nova Corumbá. The urban area of the municipality of Corumbá is represented by a GeoEye image in false-color composition RGB 432 (20/08/2012). Note: the map of Brazil (and the shapefile used to generate it) used for the elaboration of Fig 1 was extracted from the database of public domain of the Brazilian Institute of Geography and Statistics (http://mapas.ibge.gov.br/bases-e-referenciais.html).

This study was conducted in the urban area of the municipality of Corumbá, which is located in the northeastern part of the State of Mato Grosso do Sul, Brazil, in the region of the Pantanal wetland, on the border of Bolivia and adjacent to the Paraguay River (19° 00′ 33″ʹ S; 57° 39′ 12″ O; 118 m above sea level; Fig 1). The urban region of the municipality was considered to be that characterized by continuous buildings and the existence of social infrastructures for basic urban functions (e.g., housing, work, recreation, and circulation). According to the Brazilian Institute of Geography and Statistics, the municipality was estimated to be inhabited by a population of 108,010 in 2014, with a demographic density of 1.60 inhabitants/km^2^, 90% of which resided in the urban area [15].

The urban area of the municipality is located in a mountainous region known as *Morraria do Urucum*, in an area of submontane deciduous forest. The predominant vegetal coverage is the Brazilian Cerrado, which is a savannah-like biome typical of the Pantanal wetland [16].

According to the Köppen classification system, the climate of the municipality of Corumbá is tropical (Aw) and megathermal, with a dry winter and wet summer [17]. The dry season runs from April to September, and the rainy season runs from October to March [18].

### Sand Fly Collection

Sand flies were collected weekly from April 2012 to March 2014 using CDC automatic light traps. The sampling sites consisted of the peridomiciliary areas of 5 residences located in neighborhoods where at least 1 human case of visceral leishmaniasis was reported in 2011 (Fig 1). Table 1 summarizes the characteristics of each sampling site. Two traps were deployed in each sampling site between 17:00 and 07:00 the next morning. Sampling time per trap was 1,050 h, and the total sampling time was 105,000 h.

**Table 1:** General characteristics of sampling sites.

Residence (neighborhood)	General characteristics	Domesticated animals (number)
Centro (1)		
	Located in commercial center of the city; Large peridomicile area with large to medium-sized trees, some of which are fruit trees; Sampling site closest to Paraguay River (approximately 500 m).	Dogs (2) Chicken (1)
Maria Leite (2)		
	Located in northeastern outskirts of the city; Larger peridomicile area in comparison to other sites; large and medium-sized trees, some of which are fruit trees.	Dogs (2) Chickens (15^a^) Geese (5) Ducks (3)
Cristo Redentor (3)		
	Located in southeastern periphery of the city; Peridomicile area limited by the ridge of the hills covered with native vegetation, with some small trees.	Dog (1)
Popular Nova (4)		
	Located in southeastern periphery of the city; Smaller peridomicile area in comparison to the other sites; Two medium-sized fruit trees.	Dog (1)
Nova Corumbá (5)		
	Located in southern periphery of the city; Peridomicile area limited by a ridge of hills covered with native vegetation, with small and medium-sized trees, some of which are fruit trees.	Dogs (5) Chickens (4) Cats (3)

^a^The number of chickens at this residence varied throughout the study but was always greater than 15.

The specimens were identified according to the classification system of Galati [19]. Generic names were abbreviated according to Marcondes [20]. All males collected during the 2 two years, as well as females collected during the first 6 months of 2012, were slide-mounted for identification. Thereafter, collected females that did not have blood in their gut were dissected, and identified on the basis of their spermatheca characteristics. These females were then placed in 1.5-mL microtubes with isopropyl alcohol to determine the presence of *Leishmania* DNA, the results of which are described by Oliveira et al. [21].

### Vegetal Coverage and Impervious Surface Areas

From August 20, 2012, GeoEye-1 satellite images with 0.4-m resolution were used as the cartographic basis to determine the environmental variables of vegetation and impervious surface areas (ISAs), both of which are found within the urban environment. The images were ortho-rectified and geometrically corrected using a defined projection and datum. The projection used was the Universal Transverse Mercator, southern hemisphere, Zone 21 and the datum was WGS84.

These bands were combined to generate a multispectral image from which atmospheric correction could be performed to calculate NDVI, normalized difference water index (NDWI), and ISA around the sand fly collection points, with buffers of 100 and 200 m. These procedures were performed using the software PCI Geomatica 9.1 [22].

NDVI values ranged from -1 to +1, and were calculated using the following equation proposed by Rouse et al. [23]:

$$NDVI=\frac{\left(NIR-R\right)}{\left(NIR+R\right)}$$

in which *NIR* is the reflectance of vegetation in the near infrared band and *R* is the reflectance of vegetation in the red band.

The NDVI of each sampling site was stratified to obtain variables related to landscape attributes at different scales, such as habitat complexity (mean NDVI) and heterogeneity (standard deviation of NDVI) [3]. Habitat complexity is defined as the density and development of the vertical stratum in a particular unit of area, while habitat heterogeneity is the structure of the vegetation on the horizontal plane [24].

NDWI values also ranged from -1 to +1, and were calculated from the following equation proposed by McFeeters [25]:

$$NDWI=\frac{\left(GREEN-NIR\right)}{\left(GREEN+NIR\right)}$$

in which *NIR* is the reflectance of vegetation in the near infrared band and *GREEN* is the reflectance of vegetation in the green band.

ISA is the degree of impermeability of the soil, which was calculated from the equation by Carlson and Arthur [26]:

$$ISA={\left[1-{\left(\frac{\left(NDVI-NDVI_{0}\right)}{\left(NDVI_{S}+NDVI_{0}\right)}\right)}^{2}\right]}_{dev}$$

in which *NDVI*_0_ is the NDVI for exposed soil and *NDVI*_*S*_ is the NDVI for dense vegetation. The term *dev* indicates that the formula is only appropriate for regions classified as urban, with numbers closer to 1 indicative of greater impermeability.

Percent tree canopy cover (vegetal coverage) in each sampling site was estimated using a point intercept spherical densiometer. This equipment consists of a square convex mirror with 36 vertices that reflect the woody vegetation coverage in 4 directions (north, south, east, and west), with each observation occurring at 90° rotation in relation to the previous point [27]. The arithmetic mean of the data collected for all 4 directions was calculated to determine the percentage of vegetal coverage using a simple rule of 3. At each collection point, 5 random measurements were made.

### Meteorological Data

The climate data for the study period were extracted from the Mato Grosso do Sul Center for Weather, Climate, and Water Resources (www.cemtec.ms.gov.br), which is linked to the Brazilian National Meteorological Institute. Daily readings of temperature, relative air humidity, rainfall, and wind velocity were obtained. For temperature and humidity, mean daily readings were considered 7, 15, and 30 days prior to the collection date. Similar methods were used to determine rainfall; however, accumulated (sum) values, rather than mean values, were used.

### Statistical Analysis

Descriptive measures such as the geometric mean (Williams means, Mw) [28,29], arithmetic mean, median, standard deviation, minimum, and maximum were calculated to describe the total number of specimens collected and the 3 most abundant species. The hypothesis of equality of proportional distribution of the total number of specimens and the total of the 3 most abundant species at each collection site for both sexes were assessed using the Kruskal-Wallis test.

The Wilcoxon test was used for comparisons of the absolute frequencies of the total number of sand flies and of the 3 most abundant species stratified by sex and season (dry or rainy).

The association between meteorological variables and the absolute frequency of sand flies was evaluated using the Spearman correlation coefficient. The same analysis was used to measure the degree of linear relationship between the number of species observed and the environmental variables (vegetation and ISA) under study.

The analysis was conducted using R software version 3.3.0 [30] and by employing a 5% (*α* = 0.05) significance level.

### Ethical Statement

This study received the approval of the Animal Experimentation Ethics Committee of the Federal University of Mato Grosso do Sul (Brazil), under process number 491/2013. The research group has a permanent license for the collection of zoological material, issued by the Brazilian Institute of the Environment and Renewable Natural Resources (IBAMA: SISBio 25952–1). Field studies were carried out on 5 private properties, the owners of which gave permission to conduct the study in their respective peridomiciliary areas. In addition, the field studies did not involve any endangered or protected species.

## Results

A total of 750 weekly collections were performed from April 2012 to March 2014, through which 14,317 specimens of sand flies were caught: 7,370 specimens during 390 collections in the first year and 6,947 specimens during 360 collections in the second year. The specimens were distributed among 8 genera (*Brumptomyia*, *Evandromyia*, *Lutzomyia*, *Micropygomyia*, *Martinsmyia*, *Nyssomyia*, *Psathyromyia*, and *Sciopemyia*) and represented 13 species (Table 2). This study provides the first report of *Ny*. *whitmani* in the study region.

**Table 2:** Absolute frequency of sand flies according to sex, sampling site (neighborhood), and species richness of the sampling site.

Species	Sampling site										Total
	Centro		Maria Leite		Cristo Redentor		Popular Nova		Nova Corumbá		
	♂	♀	♂	♀	♂	♀	♂	♀	♂	♀	
*Br*. *brumpti*	-	-	-	-	-	-	-	-	2	2	4
*Ev*. *aldafalcaoae*	2	3	-	1	1	-	-	-	-	1	8
*Ev*. *cortelezzii*	-	1	-	1	-	1	-	2	-	2	7
*Ev*. *corumbaensis*	9	48	8	22	21	63	10	27	8	36	252
*Ev*. *sallesi*	1	5	3	7	3	3	-	2	-	2	26
*Ev*. *walkeri*	1	1	-	1	-	-	-	-	-	2	5
*Lu*. *cruzi*	3,100	548	4,004	417	1,151	233	389	100	2,996	511	13,449
*Lu*. *forattinii*	14	9	17	12	158	61	3	10	63	114	461
*Mi*. *peresi*	1	2	1	2	23	9	7	4	11	8	68
*Mt*. *oliveirai*	-	-	-	1	12	3	3	3	1	4	27
*Ny*. *whitmani*	-	-	-	-	-	-	-	-	-	1	1
*Pa*. *bigeniculata*	-	-	-	-	2	1	-	-	-	1	4
*Sc*. *sordellii*	-	-	-	1	-	1	-	1	-	2	5
**Total**	3,128	617	4,033	465	1,371	375	412	149	3,081	686	14,317
**Species richness**	8		10		10		8		13		-

Br: Brumptomyia; Ev.: Evandromyia; Lu: Lutzomyia; Mi: Micropygomyia; Mt: Martinsmyia; Ny.: Nyssomyia; Pa.: Psathyromyia; Sc.: Sciopemyia.

Table 3 presents the descriptive measures for the total number of sand flies caught and the 3 most abundant species at each collection site. In all cases (total and species analysis), the Kruskal-Wallis test revealed that the monthly arithmetic mean differed between the collection sites, with one or more sites standing out in terms of sand fly abundance. Geometric means (Williams means, Mw) demonstrated the same result. Owing to these differences, the total number of sand flies was analyzed in a conditional manner.

**Table 3:** Descriptive measures of total number of sand flies, as well as populations of *Ev*. *corumbaensis*, *Lu*. *cruzi*, and *Lu*. *forattinii*, with respect to sampling site (neighborhood).

Species	Sampling site	Arithmetic mean	Williams mean	Standard deviation	Median	Minimum	Maximum	*p*-value^a^
*Ev*. *corumbaensis* (F)								
	Centro	0.64	0.33	1.28	0	0	8	0.142
	Cristo Redentor	0.84	0.37	1.87	0	0	10	
	Maria Leite	0.29	0.17	0.67	0	0	3	
	Nova Corumbá	0.48	0.25	1.00	0	0	4	
	Popular Nova	0.36	0.20	0.86	0	0	6	
	**Total**	0.52	0.27	1.22	0	0	10	
*Ev*. *corumbaensis* (M)								
	Centro	0.12	0.07	0.43	0	0	2	0.035
	Cristo Redentor	0.28	0.17	0.58	0	0	2	
	Maria Leite	0.11	0.07	0.39	0	0	2	
	Nova Corumbá	0.11	0.07	0.39	0	0	2	
	Popular Nova	0.13	0.08	0.53	0	0	4	
	**Total**	0.15	0.09	0.47	0	0	4	
*Ev*. *corumbaensis* (MF)								
	Centro	0.76	0.36	1.50	0	0	9	0.083
	Cristo Redentor	1.12	0.48	2.20	0	0	12	
	Maria Leite	0.40	0.24	0.74	0	0	3	
	Nova Corumbá	0.59	0.30	1.13	0	0	5	
	Popular Nova	0.49	0.26	1.11	0	0	6	
	**Total**	0.67	0.33	1.44	0	0	12	
*Lu*. *cruzi* (F)								
	Centro	7.31	1.41	10.13	3	0	38	<0.001
	Cristo Redentor	3.11	0.91	5.74	1	0	40	
	Maria Leite	5.56	1.17	12.03	2	0	93	
	Nova Corumbá	6.81	1.32	12.13	3	0	65	
	Popular Nova	1.33	0.59	1.88	0	0	8	
	**Total**	4.82	1.08	9.51	1	0	93	
*Lu*. *cruzi* (M)								
	Centro	41.33	2.45	96.50	15	0	750	<0.001
	Cristo Redentor	15.35	1.55	34.94	2	0	192	
	Maria Leite	53.39	2.58	112.04	17	0	689	
	Nova Corumbá	39.95	2.36	64.65	8	0	310	
	Popular Nova	5.19	1.20	8.08	2	0	48	
	**Total**	0.00	0.00	0.00	0	0	0	
*Lu*. *cruzi* (MF)								
	Centro	48.64	2.69	103.60	22	0	787	<0.001
	Cristo Redentor	18.45	1.81	39.95	4	0	232	
	Maria Leite	58.95	2.73	117.22	18	0	696	
	Nova Corumbá	46.76	2.58	73.79	11	0	361	
	Popular Nova	6.52	1.37	9.18	4	0	53	
	**Total**	35.86	2.24	81.54	7	0	787	
*Lu*. *forattinii* (F)								
	Centro	0.12	0.06	0.57	0	0	4	<0.001
	Cristo Redentor	0.81	0.36	1.85	0	0	12	
	Maria Leite	0.16	0.10	0.49	0	0	3	
	Nova Corumbá	1.52	0.34	6.63	0	0	54	
	Popular Nova	0.13	0.08	0.47	0	0	3	
	**Total**	0.55	0.19	3.14	0	0	54	
*Lu*. f*orattinii* (M)								
	Centro	0.19	0.08	1.00	0	0	8	<0.001
	Cristo Redentor	2.11	0.62	4.45	0	0	24	
	Maria Leite	0.23	0.14	0.56	0	0	3	
	Nova Corumbá	0.84	0.37	1.82	0	0	10	
	Popular Nova	0.04	0.02	0.26	0	0	2	
	**Total**	0.68	0.24	2.33	0	0	24	
*Lu*. *forattinii* (MF)								
	Centro	0.31	0.12	1.46	0	0	12	<0.001
	Cristo Redentor	2.92	0.83	5.32	1	0	25	
	Maria Leite	0.39	0.22	0.84	0	0	4	
	Nova Corumbá	2.36	0.56	7.91	0	0	64	
	Popular Nova	0.17	0.10	0.53	0	0	3	
	**Total**	1.23	0.37	4.47	0	0	64	
Total (F)								
	Centro	8.23	1.57	10.65	4	0	39	<0.001
	Cristo Redentor	5.00	1.20	8.59	2	0	60	
	Maria Leite	6.20	1.27	12.69	2	0	98	
	Nova Corumbá	9.13	1.50	17.42	4	0	107	
	Popular Nova	1.99	0.76	2.58	1	0	12	
	Total	6.11	1.26	11.69	2	0	107	
Total (M)								
	Centro	41.71	2.48	96.52	16	0	750	<0.001
	Cristo Redentor	18.28	1.82	38.02	4	0	204	
	Maria Leite	53.77	2.60	112.64	17	0	696	
	Nova Corumbá	41.08	2.45	65.96	9	0	320	
	Popular Nova	5.49	1.25	8.40	3	0	51	
	**Total**	32.07	2.12	76.31	6	0	750	
Total (MF)								
	Centro	49.93	2.80	103.90	24	0	789	<0.001
	Cristo Redentor	23.28	2.14	44.91	7	0	264	
	Maria Leite	59.97	2.77	118.16	19	0	704	
	Nova Corumbá	50.21	2.72	79.58	18	0	427	
	Popular Nova	7.48	1.50	9.79	5	0	57	
	**Total**	38.18	2.38	83.40	10	0	789	

^a^ Kruskal-Wallis test; F = females; M = males; MF = sum of males and females; *Ev*: *Evandromyia; Lu*: *Lutzomyia*.

*Lu*. *cruzi* was the most frequently collected species, accounting for 93.94% of the total, followed by *Lu*. *forattinii* (3.22%) and *Ev*. *corumbaensis* (1.76%). This ranking of abundance was found at all collection sites. Proportionally, males were significantly more abundant than females (W = 65,797; *p* < 0.001). This proportion held true when analyzing the 3 most frequent species separately: *Lu*. *cruzi* (W = 66,444.50; *p* < 0.001), *Lu*. *forattinii* (W = 48,327.0; *p* = 0.006), and *Ev*. *corumbaensis* (W = 38,346.50; *p* < 0.001).

**Fig 2:**
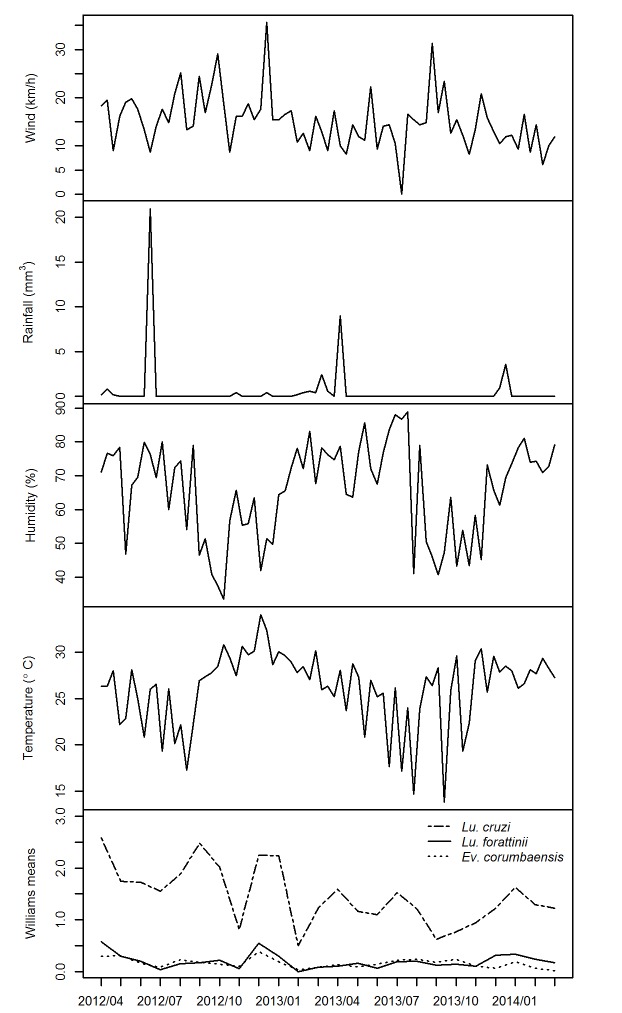
Monthly distribution of *Lu*. *cruzi*, *Lu*. *forattinii*, and *Ev corumbaensis* and the monthly arithmetic mean of the climate variables^a^ in Corumbá, Mato Grosso do sul, Brazil, between April 2012 to March 2014. ^a^ Rainfall was considered in terms of monthly accumulation.

The geometric means of monthly distribution of *Lu*. *cruzi*, *Lu*. *forattinii*, and *Ev*. *corumbaensis* and the monthly arithmetic means of the climate variables (except rainfall, which was considered in terms of monthly accumulation) are presented in Fig 2. Throughout the study period, the annual average temperature was 26.24°C, the annual average relative air humidity was 67.43%, and the accumulated rainfall was 2,312.80 mm^3^. Table 4 presents the climatic variable data. No significant association was found between the absolute frequencies (total and per species) of sand flies and meteorological variables, even after considering all assessed derivations (daily mean of the collection date and means measured at 7, 15, and 30 days prior to each collection date). In the analysis of abundance according to season, only *Ev*. *corumbaensis* females failed to confirm the hypothesis of equality between seasons (W = 503,50; *p* = 0.05). However, 4 high population peaks were found in the rainy season and 2 smaller population peaks were found in the dry season for *Lu*. *cruzi*. Likewise, 2 population peaks were found in the rainy season for both *Lu*. *forattinii* and *Ev*. *corumbaensis*.

**Table 4:** Descriptive measures of meteorological variables in Corumbá, Mato Grosso do Sul, between April 2012 to March 2014.

Variable	Arithmetic mean	Standard deviation	Median	Minimum	Maximum
**Temperature**					
Collection day	26.10	3.97	27.04	13.77	34.08
Previous 7	25.71	2.99	26.50	17.75	32.56
Previous 15	25.61	2.57	26.10	19.59	31.14
Previous 30	25.65	2.30	25.96	21.40	29.60
**Humidity**					
Collection day	65.76	14.08	69.46	33.38	89.00
Previous 7	66.61	12.21	68.55	37.55	85.54
Previous 15	66.71	11.56	69.48	36.92	85.33
Previous 30	66.68	9.94	69.53	42.47	81.81
**Rainfall**					
Collection day	0.55	2.65	0.00	0.00	21.00
Previous 7	16.27	27.46	2.80	0.00	168.00
Previous 15	26.74	35.96	11.80	0.00	170.40
Previous 30	73.11	68.99	49.40	0.00	273.00
**Wind**					
Collection day	15.16	5.67	14.76	0.00	35.64

Table 5 presents the vegetation and ISA indices obtained though remote sensing, and the percentage of vegetal coverage calculated using a spherical densiometer. No significant association was found between the absolute frequencies of sand flies and any of the studied variables, including habitat complexity and habitat heterogeneity.

**Table 5:** Descriptive measures of environmental variables in relation to sampling site, in Corumbá, Mato Grosso do Sul, Brazil.

Variable	Sampling site				
	Centro	Cristo Redentor	Maria Leite	Nova Corumbá	Popular Nova
**VC (%)**	79.34	40.83	57.90	77.34	50.83
**NDVI (buffer 100 m)**					
Mean	-0.01	0.01	-0.06	-0.02	-0.09
SD	0.23	0.18	0.18	0.17	0.18
**NDVI (buffer 200 m)**					
Mean	-0.04	-0.01	-0.07	-0.02	-0.07
Standard deviation	0.23	0.18	0.18	0.15	0.18
**NDWI (buffer 100 m)**					
Mean	-0.12	-0.18	-0.15	-0.18	-0.13
SD	0.20	0.15	0.15	0.15	0.16
**NDWI (buffer 200 m)**					
Mean	-0.11	-0.17	-0.14	-0.19	-0.13
SD	0.20	0.14	0.15	0.13	0.16
**ISA (buffer 100 m)**					
Mean	0.76	0.83	0.79	0.82	0.73
SD	0.25	0.18	0.21	0.20	0.23
**ISA (buffer 200 m)**					
Mean	0.73	0.83	0.75	0.83	0.75
SD	0.26	0.18	0.22	0.18	0.23

VC: vegetal coverage; SD: standard deviation; NDVI: normalized difference vegetation index; NDWI: normalized difference water index; ISA: impervious surface areas.

## Discussion

Although the first case of human visceral leishmaniasis was recorded in 1911 [1,31] in the Porto Esperança district of the municipality of Corumbá, studies on sand fly fauna in this city only began in the 1980s [32,33]. During this period, 12 species were identified, with *Lu*. *cruzi* and *Lu*. *forattinii* being the most abundant in urban areas [8,32–34]. *Lu*. *longipalpis* was reported in Corumbá city [35]. However, none of the subsequent studies detected this species, only recording the predominance of *Lu*. *cruzi* [8,36,37].

The vector competence of *Lu*. *cruzi* for *Leishmania* (*L*.) *infantum* has been demonstrated, and is also suspected to hold for *L*. (*L*.) *amazonensis* [38]. Furthermore, *Lu*. *forattinii* is an anthropophilic species [33] that is naturally infected by *L*. (*L*.) *infantum* in the municipality of Corumbá [36]. The presence of *Ny*. *whitmani* is now also being reported in the urban area of Corumbá. This species is associated with the transmission of *Leishmania* spp. in many regions of Brazil [39,40], and was thought to exist only at a low frequency in a rural area of Corumbá [41]. This finding underscores the need for the periodic monitoring of sand flies and studies of reservoirs of this parasite. Such studies could help identify possible population peaks of *Ny*. *whitmani* and the presence and circulation of *L*. (*Viannia*) *braziliensis* in the study area.

The appearance of a new record of species in the composition of sand fly fauna in urban areas of Corumbá and the accompanying increase in the abundance of *Lu*. *cruzi* may have occurred due to the increased of the municipality, among other factors [8]. According to Rangel and Vilela [42], environmental changes caused by human activities alter the distribution of vectors and parasites, which influence the epidemiology of leishmaniasis. Casaril et al. [8] suggested that certain factors might explain observed changes to the composition of sand fly fauna and the persistence of visceral leishmaniasis in the municipality. Such factors include deforestation, extractivist activity, the presence of rural settlements, and the disorderly occupation of hills covered with native vegetation with no planning.

The descriptive analysis of the absolute frequency of specimens showed that ecotopes in which the largest number of sand flies were captured contained chicken coops in their peridomicile areas (Tables 1 and 2). The published literature also shows that chickens attract sand flies [43–46]. Chicken coops and pigpens are recognized as resting places for adults of both sexes, where females also take their blood meals. Further, these ecotopes provide shade, moisture and soil with organic matter providing suitable conditions for breeding sites of the immature flies [47–49]. Teodoro et al. [46] showed that twice the number of sand flies (testing 8 species) could be captured in environments containing chickens compared to those without. Ximenes et al. [50] showed that the presence of domesticated animals and low cleanliness of local conditions help maintain high population densities of sand flies.

The presence of more *Lu*. *cruzi* males than females may be explained by behavioral differences feeding and copulation. It has been suggested that males are more active at searching for hosts. After finding hosts, males release sexual pheromones that attract females [51,52]. This behavior has not been recorded for *Lu*. *forattinii* or *Ev*. *corumbaensis*, despite the presence of tergal papillae on their tergites by which the sexual pheromones are released.

*Lu*. *cruzi* and *Ev*. *corumbaensis* were caught in all months of the 2-year study period. *Lu*. *forattinii* was caught in all months, except February 2013. The population peaks found in both the rainy and dry seasons demonstrate the adaptive nature of *Lu*. *cruzi* with respect to variations in the climate and urban environment. Likewise, the 2 population peaks for *Lu*. *forattinii* and *Ev*. *corumbaensis* also support this adaptive behavior. In Corumbá city, the mean monthly temperature remains higher than 25°C almost year-round. Variation in rainfall allowed the rainy and dry seasons to be clearly defined. However, relative air humidity minimally oscillated, despite having a standard deviation ranging from 14.08 to 11.56, possibly because the urban area of the municipality is adjacent to the Paraguay River. Moreover, there was a change in the hydrological regimen of this river in 2014, with the flood period, (which normally occurs from January to March) being recorded from January to June.

*Ev*. *corumbaensis* was the only species that was significantly associated with the rainy season. The abundance of this species has not been found to be related to meteorological variables in other regions of Brazil. The tendency toward greater abundance in the rainy season has been reported for *Lu*. *longipalpis* in the northeastern, central western, and southeastern regions of the country [1,4,44,53]. This trend has also been recorded for *Lu*. *cruzi* in Corumbá [33] and in the municipality of Jaciara, State of Mato Grosso, Brazil [54]. However, these insects have also been found during the driest months [55,56]. *Ev*. *corumbaensis*, the third most abundant species collected in this study, along with *Ev*. *sallesi*, *Ev*. *cortelezzii* and *Ev*. *spelunca*, forms a species complex for which human attractiveness is unknown. Further, *Ev*. *corumbaensis* not being found to be naturally infected by *Leishmania*. However, the other 3 species of the complex have been found naturally infected with *Leishmania*, or *Leishmania* DNA, in wild-caught female, has been detected [57–59]. These facts may indicate the importance of the complex in maintaining the wild cycle of *Leishmania*.

A number of studies on the seasonal distribution of insects have evaluated the relationship (either descriptively or statistically) between sand fly abundance and meteorological variables. However, most of these studies have only considered monthly mean temperature and humidity or monthly accumulated rainfall. Yet, such approaches do not always reflect the actual meteorological conditions, which may be associated with and/or exert an influence on the abundance and behavior of different arthropods. In the present study, a different approach was employed, in which the climate readings on each collection day (e.g., wind velocity) were used to assess the frequency and behavior of adults [4,60,61]. The mean values of these conditions were recorded at 7, 15, and 30 days before each collection date to evaluate factors that may influence sand flies during their immature phases and during adulthood. This approach was important because: (1) the climate variables of the micro-habitats used as breeding sites are influenced, albeit on a smaller scale, by the external environment and environmental conditions and (2) the mean development time of immature forms of *Lu*. *cruzi* ranges from 26 to 30 days in the laboratory setting [62]. Although no statistically significant associations were found with this approach, it was evident that *Lu*. *cruzi*, *Lu*. *forattinii*, and *Ev*. *corumbaensis* tend to be more abundant in the rainy season.

Studies on the diversity and distribution of species of sand flies provide key elements for clarifying the epidemiology of leishmaniasis [33,39,45]. Field studies combined with geotechnologies and different spatial analysis methods allow these insect populations to be monitored. Such approaches facilitate the identification of focal points where vector species are abundant or the identification of new potential vectors. This approach also allows the spatial distribution of reservoirs and hosts to be described. All of these parameters may be related to the presence, quantity, and type of vegetation in a given area and period of time [63,64]. The use of geotechnologies has allowed environmental characteristics to be identified in areas where visceral leishmaniasis is endemic. Such areas tend to support both human populations and large numbers of sand flies. Thus, other areas with similar environmental characteristics might represent a similar risk of *Leishmania* infecting humans and domesticated animals [3].

In the State of Mato Grosso, Brazil, a high abundance of *Lu*. *cruzi* has been found in municipalities near the Pantanal wetlands and *Cerrado* biome, suggesting that these areas are the preferred environments for these species [65]. A similar abundance has been found in the municipality of Corumbá, where the predominant vegetation cover is that of the savanna (*Cerrado*) biome typical of the Pantanal wetland [8,33,34].

In the present study, remote sensing was used to evaluate vegetation and ISAs using radiometric indices. The percentage of vegetal coverage or phytomass was measured directly in the field with a spherical densitometer. Neither method showed any significant association between total abundance or abundance by species and the quantitative indices of phytomass, wetness, and ISAs. Consequently, the landscape attributes (habitat complexity and habitat heterogeneity) determined from the NDVI did not influence on the abundance and distribution of *Lu*. *cruzi*. This result contradicts that reported for *Lu*. *longipalpis* in Campo Grande city, State of Mato Grosso do Sul [3]. However, just 5 locations were sampled in the current study, which might not be representative of the entire study area and might not reflect the relationships among these variables accurately.

Even without any correlation to species abundance, spectral indices and percentages of vegetal coverage are of considerable importance to entomology, because the analysis of vegetation allows indirect factors that influence the behavior of sand flies to be evaluated, including temperature, relative air humidity, luminosity, and altitude [66]. Andrade et al. [67] showed the importance of evaluating the wetness of vegetation using the NDWI. This is because NDWI is related to the water content of the vegetation and the soil, which is directly related to the development of immature forms of the insect, as the larval stages of sand flies require a wet environment.

This study used a GeoEye image obtained during the dry season in an area of submontane deciduous forest (which loses more than 50% of its leaves in the dry season) [16]. This approach may have influenced the values of the 3 spectral indices, particularly when sampling sites close to the hills, which contain this type of vegetation. In sampling sites where the buffers did not include mountains, the primary type of vegetation was more likely to be riparian forest, which does not lose as much of its leaf cover and is more like a sheet, or urban vegetation, which is usually arboreal. These factors may influence the abundance and diversity of sand flies and other insects. Although the use of images obtained in the dry season may have limited the study, their use in this study was justified because phytophysiognomies are more distinct in the dry period.

The present study aimed to evaluate the relationship between the abundance of medically important insects and environmental factors, including meteorological variables. Such studies should consider how interactions among these variables are related to local/individual characteristics of the analyzed ecotopes. Failure to consider such relationships could lead to inadequate or incomplete measurements and interpretations. A recent review considered on the impact of environmental, climatic, and social changes on vector-related infectious diseases [5]. This review highlighted the levels of complexity involved in describing and predicting the impact of climate changes on the transmission of infectious agents by vectors. While it is acknowledged that climate patterns directly affect the abundance of vectors and the transmission of infectious agents. However, the authors stressed that this influence may be significantly altered by non-climatic (epidemiological, environmental, social, economic, and demographic) confounding factors that camouflage the actual magnitude and spatial extension of transmissions at different scales.

The seasonal distribution of sand fly species demonstrated in the present study was primarily represented by the number of specimens of *Lu*. *cruzi* that were caught, accounting for 93.94% of the total. Monthly variation showed that *Lu*. *cruzi* exhibits considerable plasticity, with it being found in all collection months, including the dry and rainy seasons, in the municipality of Corumbá. Thus, there is a risk of infection throughout the entire year, with periods when the risk is greater. *Lu*. *cruzi*, the reconized vector of *L*. (*L*.) *infantum* and *Lu*. *forattinii*, also a probable vector of this parasite, were found in all the areas investigated. The sampling site located in Centro neighborhood presented the highest Williams mean for *Lu*. *cruzi*, reflecting both high frequencies and evenness in the collections. The sampling sites located in Nova Corumbá and Cristo Redentor neighborhoods presented the highest Williams mean for *Lu*. *forattinii*. Thus, it seems that these three areas may offer greater risk for the transmission of visceral leishmaniasis. This result underscores the need for improved planning and decision making to control visceral leishmaniasis, as well as the need to adopt environmental health education practices targeted at the local population.

## References

[1] Deane LM. Leishmaniose visceral no Brasil Estudos sobre reservatórios e transmissores realizados no Estado do Ceará. Rio de Janeiro: Serviço Nacional de Educação Sanitária; 1956.

[2] Peterson AT, Shaw J. *Lutzomyia* vectors for cutaneous leishmaniasis in Southern Brazil: ecological niche models, predicted geographic distributions, and climate change effects. Int J Parasitol. 2003; 33: 919–31. 10.1016/S0020-7519(03)00094-8 12906876

[3] Oliveira EF, Silva EA, Fernandes CES, Paranhos Filho AC, Gamarra RM, Ribeiro AA, et al Biotic factors and occurrence of *Lutzomyia longipalpis* in endemic area of visceral leishmaniasis, Mato Grosso do Sul, Brazil. Mem Inst Oswaldo Cruz. 2012; 107: 396–401. 10.1590/S0074-02762012000300015 22510836

[4] Oliveira EF, Fernandes CES, Silva EA, Brazil RP, Oliveira AG. Climatic factors and population density of *Lutzomyia longipalpis* (Lutz & Neiva, 1912) in an urban endemic area of visceral leishmaniasis in Midwest Brazil. J Vector Ecol. 2013; 38: 224–228. 10.1111/j.1948-7134.2013.12034.x 24581349

[5] Parham PE, Waldock J, Christophides GK, Hemming D, Agusto F, Evans KJ, et al Climate, environmental and socio-economic change: weighing up the balance in vector-borne disease transmission. Philos Trans R Soc Lond B Biol Sci. 2015; 370: pii. 20130551 10.1098/rstb.2013.0551 25688012 PMC4342957

[6] Peterson AT, Sánchez-Cordero V, Beard CB, Ramsey JM. Ecologic niche modeling and potential reservoirs for Chagas disease, Mexico. Emerg Infect Dis. 2002; 8: 662–667. 10.3201/eid0807.010454 12095431 PMC2730326

[7] Werneck GL, Costa CHN, Walker AM, David JR, Wand M, Maguire JH. Multilevel modelling of the incidence of visceral leishmaniasis in Teresina, Brazil. Epidemiol Infect. 2007; 135: 195–201. 10.1017/S0950268806006881 16824254 PMC2870576

[8] Casaril AE, Monaco NZN, Oliveira EF, Eguchi GU, Paranhos Filho AC, Pereira LE, et al Spatiotemporal analysis of sandfly fauna (Diptera: Psychodidae) in an endemic area of visceral leishmaniasis at Pantanal, central South America. Parasit Vectors. 2014; 7: 364 10.1186/1756-3305-7-364 25128480 PMC4261527

[9] Câmara G, Monteiro AMV. Conceitos básicos em ciência da geoinformação In: Câmara G, Davis C, Monteiro AMV, editors. Introdução à ciência da geoinformação. São José dos Campos: INPE; 2001 pp. 12–41.

[10] Novo EMLM. Sensoriamento remoto: princípios e aplicações. 3rd ed São Paulo: Edgard Blücher; 2008.

[11] Cross ER, Newcomb WW, Tucker CJ. Use of weather data and remote sensing to predict the geographic and seasonal distribution of. *Phlebotomus papatasi* in southwest Asia. Am J Trop Med Hyg. 1996; 54: 530–536. 8644911 10.4269/ajtmh.1996.54.530

[12] Elnaiem DA, Connor SJ, Thomson MC, Hassan MM, Hassan HK, Aboud MA, et al Environmental determinants of the distribution of *Phlebotomus orientalis* in Sudan. Ann Trop Med Parasitol. 1998; 92: 877–887. 10.1080/00034989858925 10396348

[13] Fernández MS, Salomón OD, Cavia R, Perez AA, Acardi SA, Guccione JD. *Lutzomyia longipalpis* spatial distribution and association with environmental variables in an urban focus of visceral leishmaniasis, Misiones, Argentina. Acta Trop. 2010; 114: 81–87. 10.1016/j.actatropica.2010.01.008 20096256

[14] Özbel Y, Balcioğlu C, Ölgen MK, Şimsek FM, Töz SÖ, Ertabaklar H, et al Spatial distribution of phlebotomine sand flies in the Aydin Mountains and surroundings: the main focus of cutaneous leishmaniasis in western Turkey. J Vector Ecol. 2011; 36: S99–S105. 10.1111/j.1948-7134.2011.00118.x 21366787

[15] Brazilian Institute of Geography and Statistics (Instituto Brasileiro de Geografia e Estatística—IBGE). Estimativas da população residente: Corumbá, Mato Grosso do Sul 2014 Available: http://cod.ibge.gov.br/36WA. Accessed 12 January 2016.

[16] Mato Grosso do Sul. Atlas Multirreferencial. Campo Grande: Secretaria de Planejamento e Coordenação Geral (SEPLAN); 1990.

[17] Alvares CA, Stape JL, Sentelhas PC, Gonçalves JLM, Sparovek G. Köppen’s climate classification map for Brazil. Meteorol Z. 2013; 22: 711–728. 10.1127/0941-2948/2013/0507

[18] Soriano BMA. Caracterização climática de Corumbá, MS. Corumbá: Embrapa-CPAP, 1997.

[19] Galati EAB. Classificação de Phlebotominae In: Rangel EF, Lainson R, editors. Flebotomíneos do Brasil. Rio de Janeiro: Fiocruz; 2003 pp. 23–51.

[20] Marcondes CB. A proposal of generic and subgeneric abbreviations for phlebotomine sandflies (Diptera: Psychodidae: Phlebotominae) of the world. Entomol News. 2007; 118: 351–356. 10.3157/0013-872X(2007)118[351:APOGAS]2.0.CO;2

[21] Oliveira EF, Casaril AE, Mateus NLF, Murat PG, Fernandes WS, Oshiro ET, et al *Leishmania amazonensis* DNA in wild females of *Lutzomyia cruzi* (Diptera: Psychodidae) in the state of Mato Grosso do Sul, Brazil. Mem Inst Oswaldo Cruz. 2015; 110: 1051–1057. 10.1590/0074-02760150317 26602870 PMC4708026

[22] PCI Geomatics. PCI Geomatica 9.1 for Windows. Ontário, Canadá; 2003.

[23] Rouse JW, Haas RH, Schell JA, Deeering DW. Monitoring vegetation systems in the Great Plains with ERTS (Earth Resources Technology Satellite). In: Fraden SC, Marcanti EP, Becker MA, editors. Third ERTS-1 Symposium. Washington DC: NASA; 1974. pp. 309–317.

[24] Dajoz R. Princípios de ecologia. 7th ed Porto Alegre: Artmed; 2005.

[25] McFeeters SK. The use of the Normalized Difference Water Index (NDWI) in the delineation of open water features. Int J Remote Sens. 1996; 17: 1425–1432. 10.1080/01431169608948714

[26] Carlson TN, Arthur ST. The impact of land use—land cover changes due to urbanization on surface microclimate and hydrology: a satellite perspective. Glob Planet Change. 2000; 25: 49–65. 10.1016/S0921-8181(00)00021-7

[27] Lemmon PE. A spherical densiometer for estimating forest overstory density. For Sci. 1956; 2: 314–320.

[28] Haddow AJ. Studies on the biting habits and medical importance of east African mosquitoes in the genus *Aedes*. I. Subgenera *Aedimorphus*, *Banksinella* and *Nunnius*. Bull Entomol Res. 1960; 50: 759–779.

[29] Haddow AJ. Studies on the biting habits of African mosquitoes: an appraisal of methods employed, with special reference to the twenty-four-hour catch. Bull Entomol Res. 1954; 45: 199–242.

[30] R Core Team. R: A language and environment for statistical computing. R Foundation for Statistical Computing, Vienna, Austria; 2016.

[31] Migone LE. Un caso de Kalazar a Assuncion (Paraguay). Bull Soc Pathol Exot. 1913; 6: 118–120.

[32] Galati EAB, Rego FA Junior, Nunes VLB, Oshiro ET. Fauna flebotomínica do município de Corumbá, Mato Grosso do Sul, Brasil e descrição de *Lutzomyia forattinii*, sp. n. (Diptera, Psychodidae, Phlebotominae). Rev Bras Entomol. 1985; 29(2): 261–266.

[33] Galati EAB, Nunes VLB, Rego FA Junior, Oshiro ET, Rodrigues M. Estudo de flebotomíneos (Diptera, Psychodidae) em foco de leishmaniose visceral no Estado de Mato Grosso do Sul, Brasil. Rev Saude Publica. 1997; 31(4): 378–390. 10.1590/S0034-89101997000400007 9595767

[34] Santos SO, Arias J, Ribeiro AA, Hoffmann MP, Freitas RA, Malacco MAF. Incrimination of *Lutzomyia cruzi* as a vector of American visceral leishmaniasis. Med Vet Entomol. 1998; 12(3): 315–317. 10.1046/j.1365-2915.1998.00104.x 9737605

[35] Santos SO, Arias J, Hoffmann MP, Furlan MBG, Ferreira WF, Pereira C, et al The presence of *Lutzomyia longipalpis* in a focus of American Visceral Leishmaniasis where the only proven vector is *Lutzomyia cruzi*. Corumbá, Mato Grosso do Sul state. Rev Soc Bras Med Trop. 2003; 36: 633–634. 10.1590/S0037-86822003000500017 14576882

[36] Pita-Pereira D, Cardoso MAB, Alves CR, Brazil RP, Britto C. Detection of natural infection in *Lutzomyia cruzi* and *Lutzomyia forattinii* (Diptera: Psychodidae: Phlebotominae) by *Leishmania infantum chagasi* in an endemic area of visceral leishmaniasis in Brazil using a PCR multiplex assay. Acta Trop. 2008; 107(1): 66–69. 10.1016/j.actatropica.2008.04.015 18502392

[37] Almeida PS, Nascimento JC, Ferreira AD, Minzão LD, Portes F, Miranda AM, et al Species of phlebotomines (Diptera, Psychodidae) collected in urban municipalities with transmission of visceral leishmaniasis in Mato Grosso do Sul State, Brazil. Rev Bras Entomol. 2010; 54: 304–310. 10.1590/S0085-56262010000200014

[38] Oliveira EF, Oshiro ET, Fernandes WS, Ferreira AMT, Oliveira AG, Galati EAB. Vector competence of *Lutzomyia cruzi* naturally demonstrated for *Leishmania infantum* and suspected for *Leishmania amazonenses*. Am J Trop Med Hyg. 2016; In press. 10.4269/ajtmh.16-0191 PMC5239689 28077746

[39] Lainson R, Shaw JJ. New World Leishmaniasis In: Cox FEG, Wakelin D, Gillespie SH, Despommier DD, editors. Topley & Wilson's Microbiology and Microbial Infections: parasitology. 10th ed London: Hodder Arnold ASM Press; 2005 pp. 313–49.

[40] de Souza AAA, dos Santos TV, Jennings YLL, Ishikawa EAY, Barata ID, Silva MGS, et al Natural *Leishmania* (*Viannia*) spp. infections in phlebotomine sand flies (Diptera: Psychodidae) from the Brazilian Amazon region reveal new putative transmission cycles of American cutaneous leishmaniasis. Parasite. 2016; 23: 22 10.1051/parasite/2016022 27235194 PMC4884270

[41] Braga-Miranda LC, Miranda M, Galati EAB. Phlebotomine fauna in a rural area of the Brazilian Pantanal. Rev Saude Publica. 2006; 40: 324–326. 10.1590/S0034-89102006000200021 16583046

[42] Rangel EF, Vilela ML. *Lutzomyia longipalpis* (Diptera, Psychodidae, Phlebotominae) and urbanization of visceral leishmaniasis in Brazil. Cad Saude Publica. 2008; 24: 2948–2952. 10.1590/S0102-311X2008001200025 19082287

[43] Alexander B, Carvalho RL, McCallum H, Pereira MH. Role of the domestic chicken (*Gallus gallus*) in the epidemiology of urban visceral leishmaniasis in Brazil. Emerg Infect Dis. 2002; 12: 1480–1485. 10.3201/eid0812.010485 12498667 PMC2738513

[44] Jeraldo VLS, Góes MAO, Casanova C, Melo CM, Araújo ED, Brandão-Filho SP, et al Sandfly fauna in an area endemic for visceral leishmaniasis in Aracaju, State of Sergipe, Northeast Brazil. Rev Soc Bras Med Trop. 2012; 45: 318–322. 10.1590/S0037-86822012000300008 22760129

[45] Lainson R, Rangel EF. *Lutzomyia longipalpis* and the eco-epidemiology of American visceral leishmaniasis, with particular reference to Brazil: a review. Mem Inst Oswaldo Cruz. 2005; 100(8): 811–827. S0074-02762006000800008. 16444411 10.1590/s0074-02762005000800001

[46] Teodoro U, Lonardoni MVC, Silveira TGV, Dias AC, Abbas M, Alberton D, et al Luz e galinhas como fatores de atração de *Nyssomyia whitmani* em ambiente rural, Paraná, Brasil. Rev Saude Publica. 2007; 41: 383–388. 10.1590/S0034-89102007000300009 17515991

[47] Aguiar GM, Medeiros WM. Distribuição regional e habitats das espécies de flebotomíneos do Brasil In: Rangel EF, Lainson R, editors. Flebotomíneos do Brasil. Rio de Janeiro: Fiocruz; 2003 pp. 207–255.

[48] Campbell-Lendrum DH, Brandão-Filho SP, Ready PD, Davies CR. Host and/or site loyalty of *Lutzomyia whitmani* (Diptera: Psychodidae) in Brazil. Med Vet Entomol. 1999; 13: 209–211. 10.1046/j.1365-2915.1999.00169.x 10484168

[49] Oliveira EF, Silva EA, Casaril AE, Fernandes CES, Paranhos Filho AC, Gamarra RM, et al Behavioral aspects of *Lutzomyia longipalpis* (Diptera: Psychodidae) in urban area endemic for visceral leishmaniasis. J Med Entomol. 2013; 50: 277–284. 10.1603/ME12082 23540114

[50] Ximenes MFFM, Castellon EG, Souza MF, Menezes AAL, Queiroz JW, Silva VPM, et al Effect of abiotic factors of seasonal population dynamics of *Lutzomyia longipalpis* (Diptera: Psychodidae) in Northeastern Brazil. J Med Entomol. 2006; 43: 990–995. 10.1093/jmedent/43.5.990. 17017238

[51] Nascimento BWL, Saraiva L, Teixeira Neto RG, Meira PCLS, Sanguinette CC, Tonelli GB, et al Study of sand flies (Diptera: Psychodidade) in visceral and cutaneous leishmaniasis areas in central western of Minas Gerais state–Brazil. Acta Trop. 2013; 125: 262–268. 10.1016/j.actatropica.2012 23178219

[52] Brazil RP, Brazil BG. Biologia de flebotomíneos neotropicais In: Rangel EF, Lainson R, editors. Flebotomíneos do Brasil. Rio de Janeiro: Fiocruz; 2003 pp. 257–274.

[53] Souza CM, Pessanha JE, Barata RA, Monteiro EM, Costa DC, Dias ES. Study on Phlebotomine sand fly (Diptera: Psychodidae) fauna in Belo Horizonte, State of Minas Gerais, Brazil. Mem Inst Oswaldo Cruz. 2004; 99: 795–803. 10.1590/S0074-02762004000800003 15761593

[54] Brito VN, Almeida ABPF, Nakazato L, Duarte R, Souza CO, Sousa VRF. Phlebotomine fauna, natural infection rate and feeding habits of *Lutzomyia cruzi* in Jaciara, state of Mato Grosso, Brazil. Mem Inst Oswaldo Cruz. 2014; 109: 899–904. 10.1590/0074-0276140112 25410993 PMC4296494

[55] Galati EAB, Nunes VLB, Dorval MEC, Oshiro ET, Cristaldo G, Espíndola MA, et al Estudo dos flebotomíneos (Diptera, Psychodidae), em área de leishmaniose tegumentar, no Estado de Mato Grosso do Sul, Brasil. Rev Saude Publica. 1996; 30: 115–128. 10.1590/S0034-89101996000200002 9077009

[56] Zeledón R, Murillo J, Gutierrez H. Ecology of *Lutzomyia longipalpis* (Lutz & Neiva, 1912) and possibilities of the existence of visceral leishmaniasis in Costa Rica. Mem Inst Oswaldo Cruz. 1984; 79: 455–459. 10.1590/S0074-02761984000400010 6533420

[57] Carvalho GMdL, Brazil RP, Saraiva L, Quaresma PF, Botelho HA, Ramos MCdNF, et al Hourly Activity and Natural Infection of Sandflies (Diptera: Psychodidae) Captured from the Aphotic Zone of a Cave, Minas Gerais State, Brazil. PLOS ONE. 2012; 7(12): e52254 10.1371/journal.pone.0052254 23284957 PMC3526590

[58] Carvalho GM, Andrade Filho JD, Falcao AL, Rocha Lima AC, Gontijo CM. Naturally infected Lutzomyia sand flies in a Leishmania-endemic area of Brazil. Vector Borne Zoonotic Dis. 2008; 8: 407–14. 10.1089/vbz.2007.0180 18429695

[59] Saraiva L, Carvalho GM, Gontijo CM, Quaresma PF, Lima AC, Falcão AL, et al Natural infection of Lutzomyia neivai and Lutzomyia sallesi (Diptera: Psychodidae) by Leishmania infantum chagasi in Brazil. J Med Entomol. 2009; 46: 1159–1163. 10.1603/033.046.0525 19769049

[60] Garms R, Walsh JF, Davies JB. Studies on the reinvasion of the onchocerciasis control programme in the Volta River Basin by *Simulium damnosum* s.I. with emphasis on the south-western areas. Tropenmed Parasitol. 1979; 30: 345–362. 575581

[61] Kakitani I, Ueno HM, Forattini OP. Parity and wind impact on the frequency of *Anopheles marajoara* in Brazil. Rev Saude Publica. 2003; 37: 280–284. 10.1590/S0034-89102003000300003 12792676

[62] Oliveira EF, Fernandes WS, Oshiro ET, Oliveira AG, Galati EAB. Alternative Method for the Mass Rearing of Lutzomyia (Lutzomyia) cruzi (Diptera: Psychodidae) in a Laboratory Setting. J Med Entomol. 2015; 52: 925–931. 10.1093/jme/tjv102 26336242

[63] Aparicio C, Bitencourt MD. Modelagem espacial de zonas de risco da leishmaniose tegumentar americana. Rev Saude Publica. 2004; 38: 511–516. 10.1590/S0034-89102004000400005 15311290

[64] Beck LR, Lobitz BM, Wood BL. Remote sensing and human health: new sensors and new opportunities. Emerg Infect Dis. 2000; 6: 217–227. 10.3201/eid0603.000301 10827111 PMC2640871

[65] Missawa NA, Lima GBM. Distribuição espacial de *Lutzomyia longipalpis* (Lutz & Neiva, 1912) e *Lutzomyia cruzi* (Mangabeira, 1938) no Estado de Mato Grosso. Rev Soc Bras Med Trop. 2006; 39(4): 337–340. 10.1590/S0037-86822006000400004 17119747

[66] Aparicio C. Utilização de geoprocessamento e sensoriamento remoto orbital para análise espacial de paisagem com incidência de leishmaniose tegumentar americana. M.Sc. Thesis, University of São Paulo. 2001. Available: http://www.teses.usp.br/teses/disponiveis/41/41134/tde-16062002-111445/pt-br.php.

[67] Andrade ARO, Silva BAK, Cristaldo G, Andrade SMO, Paranhos Filho AC, Ribeiro AA, et al Spatial distribution and environmental factors associated to phlebotomine fauna in a border area of transmission of visceral leishmaniasis in Mato Grosso do Sul, Brazil. Parasit Vectors. 2014; 7: 260 10.1186/1756-3305-7-260 24898032 PMC4055399

